# Case report: Exotropia in waardenburg syndrome with novel variations

**DOI:** 10.3389/fgene.2022.969680

**Published:** 2022-09-02

**Authors:** Lijuan Huang, Maosheng Guo, Ningdong Li

**Affiliations:** ^1^ Department of Ophthalmology, The Second Affiliated Hospital of Fujian Medical University, Quanzhou, China; ^2^ Department of Ophthalmology, Beijing Children’s Hospital, Capital Medical University, Beijing, China; ^3^ Key Laboratory of Major Diseases in Children, Ministry of Education, Beijing, China; ^4^ Department of Ophthalmology, Children’s Hospital, Capital Institute of Pediatrics, Beijing, China

**Keywords:** waardenburg syndrome, exotropia, PAX3, SOX10, COL11A2

## Abstract

**Background:** Waardenburg syndrome (WS) is a rare genetic disorder characterized by congenital sensorineural hearing loss and pigmentary abnormalities of the hair, skin and eyes. However, exotropia is rarely reported. The purpose of this study is to describe the clinical characteristics of three sporadic patients with WS and congenital exotropia and to investigate the disease-causing genes for them.

**Methods:** Patients underwent detailed physical and ocular examinations. Ocular alignment and binocular status were evaluated. DNA was extracted and whole exome sequencing was performed to detect the pathogenic variations in the disease-causing genes for WS. Cloning sequencing was carried out for those indel variations.

**Results:** Three unrelated patients were diagnosed with Waardenburg syndrome and congenital exotropia. Four novel variants, including c.136delA (p.I46Sfs*64) and c.668G>T (p.R223L) in *PAX3,* c.709dupC (p.Q237Pfs*119) in *COL11A2,* c.426G>A (p.W142X) in *SOX10* gene, were detected in this study.

**Conclusion:** Simultaneous presence of congenital exotropia and WS in our patients is suggested that WS could be involved in malfunction in the multiple nerve systems. Our genetic study will expand the mutation spectrum of *PAX3*, *COL11A2* and *SOX10* genes, and is helpful for further study on the molecular pathogenesis of WS.

## Introduction

Waardenburg syndrome (WS) is characterized by congenital sensorineural hearing loss and pigmentary abnormalities of the hair, skin and eyes, and can be inherited as an autosomal dominant or an autosomal recessive trait, with an estimated prevalence of 1/42,000 in the global population (Read and Newton, 1997). With the exceptions of sensorineural hearing loss and pigmentary abnormalities, many other clinical features are reported as dystopia canthorum, broad nasal root, bushy eyebrows with synophrys, and abnormalities in the limb, neurologic and visceral organs (Farrer et al., 1992; Gowda and Srinivasan, 2020). Based on the clinical features and molecular diagnosis, WS may be classified into 1–4 four subtypes (Ahmed jan et al., 2022).

WS type 1 (WS1, OMIM193500) is inherited as an autosomal dominant trait and caused by mutations of the *PAX3* gene. WS type 2 (WS2) is caused by mutations of *MITF* (OMIM 193510), *SNAI2* (OMIM 608890) and *SOX10* (OMIM 611584) genes (Somashekar et al., 2019; Huang et al., 2021). Patients with WS2 do not have dystopia canthorum, which is distinguished from WS1. WS type 3 (WS3, OMIM 148820) is caused by the *PAX3* mutations as well, and is distinguished from WS1 by abnormalities of the upper limb. WS type 4 (WS4, OMIM 277580), also known as Waardenburg-Shah syndrome, is usually associated with congenital megacolon and other gastrointestinal malformations. Mutations of *EDNRB* (OMIM 277580), *EDN3* (OMIM 613265) and *SOX10* genes have been reported to be responsible for WS4 (Touraine et al., 2000; Verheij and Hofstra, 2016).

Exotropia (XT) is a common form of strabismus with an estimated prevalence of 1% in the population (Govindan et al., 2005). Its etiology is not clear. It is suggested that exotropia could be the interactive results between dynamic and static factors (Von Noorden, 2002). Dynamic factors refer to those innervational factors that tend to maintain ocular alignment through establishing equilibrium between convergence and divergence. Static factors refer to those anatomical factors consisting of orbital structure, anatomy of extraocular muscles and muscle pulleys. XT can be congenital or acquired, determined by the onset of age before or after 6 months.

In the previous reports, exotropia is rarely documented as one of clinical features of Waardenburg syndrome (Dastur et al., 1995). Here, we describe the clinical features of three patients with WS and exotropia, and identify the disease-causing gene variants for these patients.

## Case presentation

### Clinical features

**FIGURE 1:**
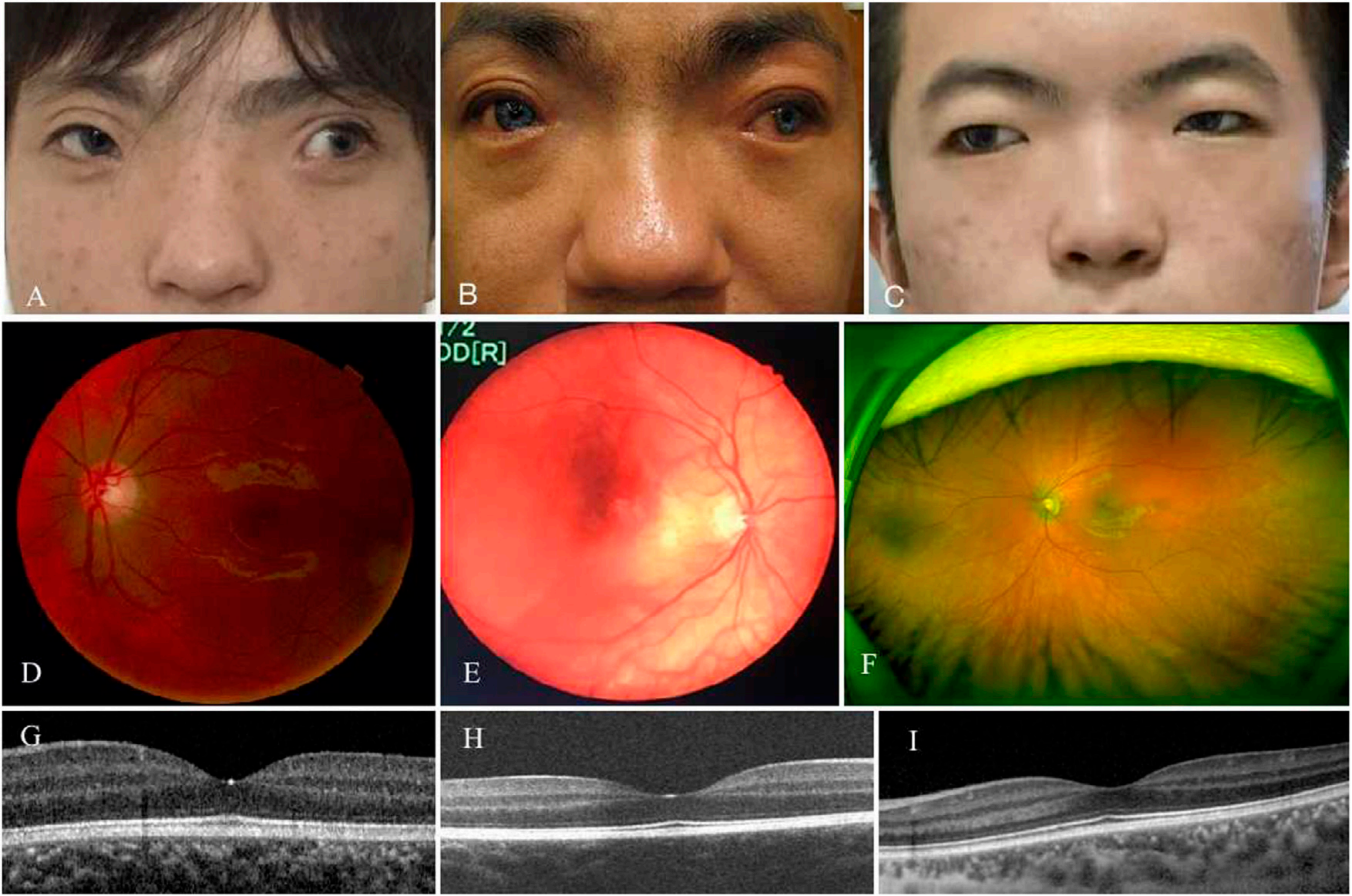
Phenotypes of three patients with WS and exotropia. All patients had an large angle exotropia **(A–C)**. They were diagnosed as WS by dystopia canthorum, broad nasal root, synophrys **(A–C)**, and hypopigmented fundus **(D–F)**. Their fovea structure were normal **(G–I)**.

**FIGURE 2:**
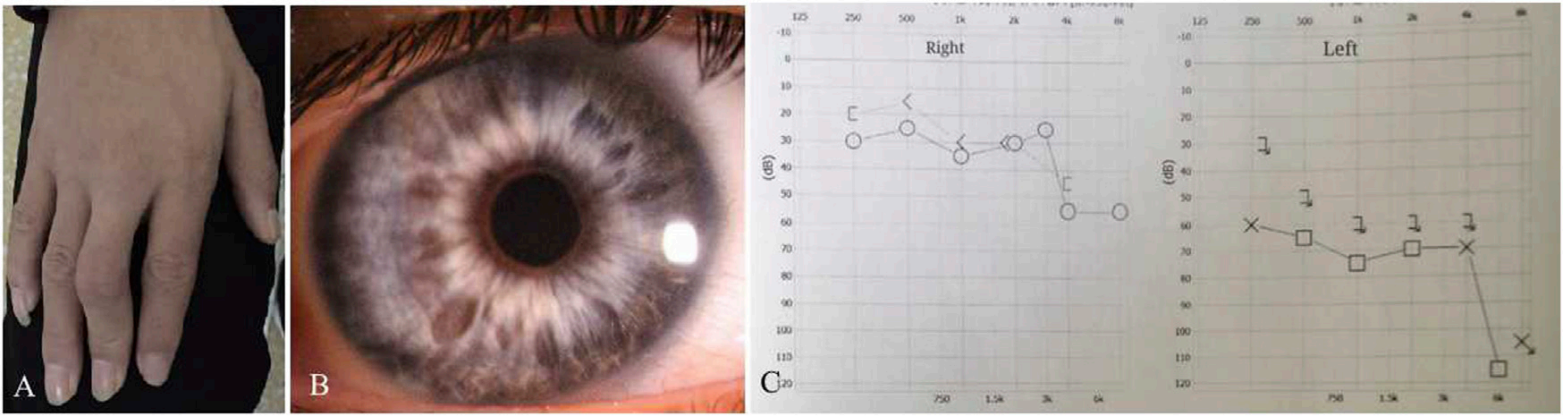
Clinical features of Patient 01. He had deformity of the fingers **(A)** and heterochromia irides **(B)**. The audiograms showed that his hearing was severely reduced on the left more than the right side **(C)**. *X*-axis, frequency in hertz (Hz); *Y*-axis, hearing level in decibels (dB).

Patient 01 from Family 01 was a 22-year old male. Medical history was negative for family history, brain injury, prematurity or hypoxia at his delivery. He had developed a constant exotropia at the age of 4 months but had not undergone surgical treatment for his strabismus. He showed a large angle exotropia of more than 45° in primary gaze position (Figure 1A). He was diagnosed as Waardenburg syndrome type 3 based on his dystopia canthorum, broad nasal root, synophrys, heterochromia irides, hypopigmented fundus appearance, normal fovea and malformed fingers, and hearing loss (Figures 1A, D, G; Figures 2A–C). His best-corrected visual acuity was 20/20 in the right eye and 16/20 in the left eye. He did not have binocular vision assessed through the Bagolini striated glasses and fixated alternatively with each of his eyes. Neurological and mental disorders were excluded by a neurologist.

Patient 02 from Family 02 was a 31-year old male. He had a notable appearance of a bald head, exotropia, broad nasal root, blue color irides, and bushy eyebrows with synophrys on his face (Figure 1B). Medical history showed that he was normally delivered without brain injury, hypoxia or prematurity at birth. He had exotropia at the age of 4∼5 months. His exotropia was intermittent before the age of 5 years, and progressively developed to a constant exotropia with growth. He never underwent strabismus surgery before visiting our clinic. He complained that his “blue eyes” came from his father. Ocular examination showed that his best-corrected visual acuity was 20/20 in the right eye and 10/20 in the left eye. He had hypopigmented fundus appearance as well (Figure 1E) and normal fovea pit (Figure 1H). Examinations of ocular alignment revealed that he had a constant exotropia with a large deviation angle of 100 prism diopter (PD) at near and distance. Prior to genetic testing, diagnosis of Waardenburg syndrome type 1 was considered according to his synophrys, broad nasal root, pigmentary disturbance of iris, hypopigmented fundus, and hearing loss in his left ear. Neurologic and visceral abnormalities were excluded after physical examinations.

Patient 03 from Family 03 was a 19-year-old male and was not found to have neurological or mental disorders or family history in his medical records. He developed a constant exotropia at the age of 5 months, and presented as a large angle exotropia of more than 35° in primary gaze position, without binocular vision and fixating alternatively with each of his eyes. He was diagnosed as Waardenburg syndrome type 1 in terms of his dystopia canthorum, broad nasal root, synophrys, depigmented irides, and hypopigmented fundus (Figures 1C,F,I). His best-corrected visual acuity was 18/20 in both eyes.

### Genetic analysis

**FIGURE 3:**
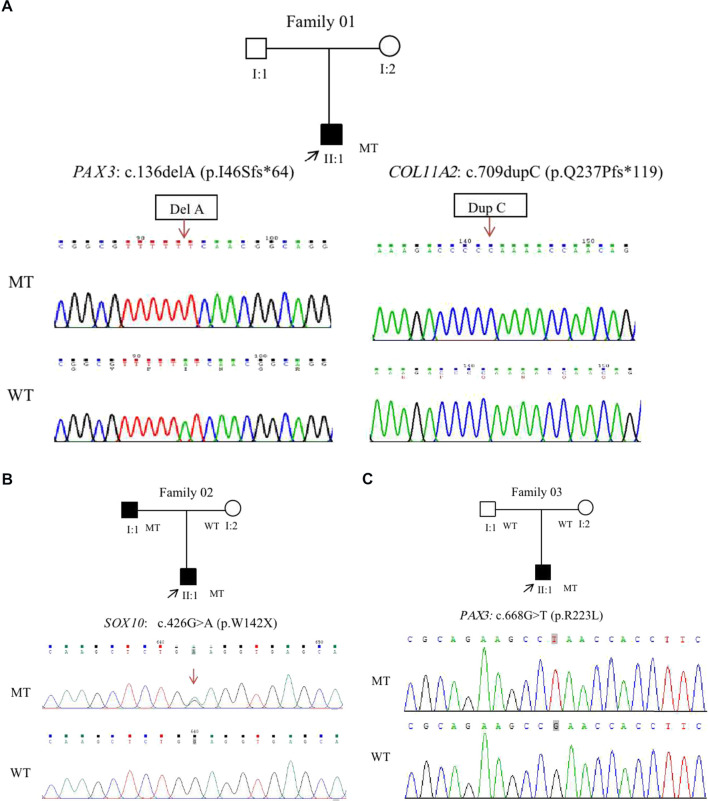
Four novel variants were identified from three WS patients in this study. A double gene variant in the *PAX3* and *COL11A2* was detected in Patient 01. Cloning sequencing showed that a single nucleotide deletion of c.136del A was detected in the *PAX3*, and a single nucleotide duplication of c.709dupC was found in the *COL11A2* gene **(A)**. A heterozygote missense variation of c.426G>A in *SOX10* was detected in the Patient 02 **(B)**. This variant resulted in an amino acid of Tryptophan (W) at Codon 142 replaced by a premature terminate codon. A novel heterozygote missense variation of c.668G>T in *PAX3* was detected in Patient 03 **(C)**. The variant led to an amino acid of Arginine at Codon 223 replaced by Leucine. Mutant type (upper row), wide type (lower row); the arrows denote the locations of the indel mutations.

**FIGURE 4:**
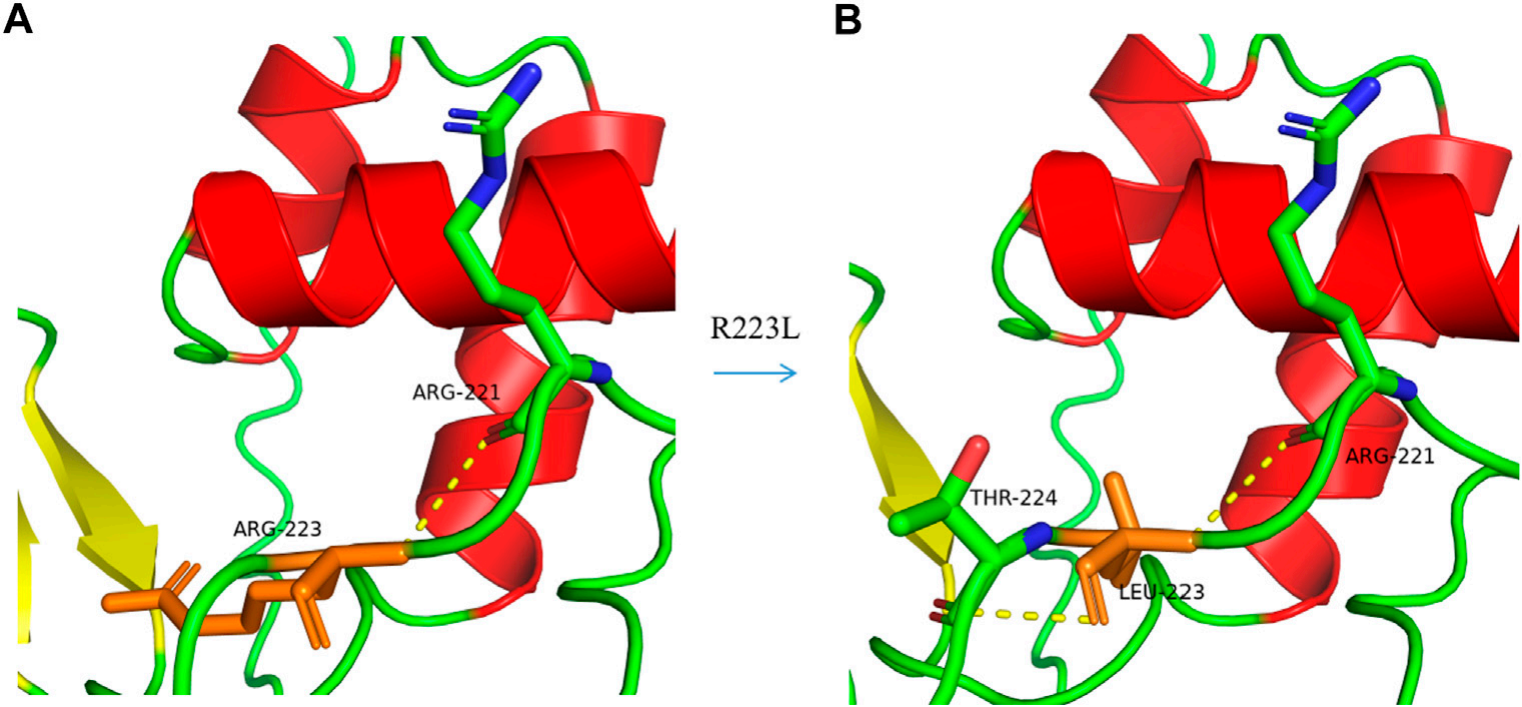
A 3-D model construction showed that the novel missense variation of c .668G>T in *PAX3* resulted in a large and positively charged amino acid of Arginine (R) at codon 223 being replaced by small and uncharged nonpolar amino acid of Leucine (L). The wild-type Arginine formed a hydrogen bond with R221 **(A)**, while Leucine could form hydrogen bonds with R221 and T224 **(B)**, which would damage the stability of the protein structure and function.

Genetic testing revealed that a double gene variation was detected in Patient 01 who carried the pathogenic variations of c.136delA (p.Ile46Serfs*64) in exon2 in *PAX3* and c.709dupC (p.Gln237Profs*119) in exon5 in *COL11A2*. Both of these variations were novel identified in this study, and predicted to produce an abnormal mRNA with a premature termination codon (PTC), which would be probably degraded due to the nonsense mediated decay (NMD) mechanism (Figure 3A). A novel heterozygous variation of c.426G>A (p.Trp142X) in the *SOX10* gene was detected in Patient 02 (Figure 3B). This variation would lead to a wild-type amino acid of Tryptophan at Codon 142 replaced by a premature termination codon, which would be degraded due to NMD mechanism. The novel identified pathogenic variation of c.668G>T (p.Arg223Leu) in *PAX3* (Figure 3C) was detected in Patient 03. A positive polar charged Argnine at codon 223 replaced by a nonpolar Leucine would be predicted to damage the stability of the protein structure and function, which was proven in the 3-D model construction using the PyMOL program (Figure 4). All above four variations were not detected in 100 ethnically matched control individuals through the Sanger sequencing. The pathogenicity assessment was listed in Table 1, based on the ACMG guidelines.

**TABLE 1:** Summary of Detected WS-related Novel Variants in this Study.

Gene	Nucleotide	Protein	Sift	Polyphen-2	CADD	ACMG	Evidence levels	SpliceAI
*PAX3*	c.136delA	p.Ile46Serfs*64	-	-	-	LP	PVS1+PM2	-
*PAX3*	c.668G>T	p.Arg223Leu	D	P	35	VUS	PM5+PM2 +PP3	N/E
*COL11A2*	c.709dupC	p.Gln237Profs*33	-	-	-	LP	PVS1+PM2	-
*SOX10*	c.426G>A	p.Trp142*	-	-	-	LP	PVS1+PM2	-

Abbreviation: d-Disease causing; P-Probably damaging; LP-Likely pathogenic; N/E-No effect.

## Discussion

In this study, we have described the clinical features of three patients with WS and large exotropia. The most prominent characteristic of our patients was their large angle congenital exotropia. Previous studies showed that few patients with WS had strabismus, and of those patients esotropia was common but exotropia was rare (Delleman and Hageman, 1978; Tang et al., 2020). In one of the reports, five of 26 patients (19%) with WS had esotropia (Delleman and Hageman, 1978). However, only two patients were described with mild exotropia in two separated reports (Dastur et al., 1995). To our knowledge, congenital exotropia with a large angle deviation is reported for the first time in our patients with WS.

Waardenburg syndrome (WS) is believed to be caused by anomalies in neural crest cell development and migration, which results in abnormal distribution of the NSC-derived melanocytes and produces an abnormal color in the hair and iris (Huang et al., 2021). In addition, another significant feature of WS is hearing loss caused by acoustic nerve injury (Boudewyns et al., 2020). As a company symptom, exotropia occurs in all of our WS patients. However, we are not sure the exact cause of their exotropia. We assume that it could be caused by abnormal nerve development due to anomalies in neural crest cell development and migration, based on the fact that congenital exotropia is usually associated with neurological disorders, craniofacial anomalies, and cerebral palsy (Choi and Kim, 2013; Lueder and Galli, 2018). In addition, recent studies demonstrate that melanin is involved in nerve development. Melanin metabolic disorders such as oculocutaneous albinism (OCA) and ocular albinism (OA) usually present as reduced visual acuity, strabismus and nystagmus (Federico and Krishnamurthy, 2022).

With the exception of exotropia, our patients present clinical and genetic heterogeneity with clinical manifestations and genotypes that are not completely consistent. Patient 01 is presumed to have WS3 because of the deformity of his fingers. He carries a double mutation of *PAX3* and *COL11A2*. Mutations of the *PAX3* gene have been recognized to be the underlying molecular pathogenesis of WS1 and WS3. As a member of the paired box family of transcription factors, *PAX3* plays an important role in neural development and myogenesis during the fetal period (Boudjadi et al., 2018). Mutations of *PAX3* have been proven to be associated with hearing loss (Kim et al., 2014). *COL11A2* gene is a protein-coding gene, playing an important role in fibrillogenesis (Vázquez-Villa et al., 2015). Mutations of *COL11A2* result in deafness in patients with DFNA13 (non-syndromic sensorineural deafness type 13) (Kunst et al., 2000). To our knowledge, hearing loss caused by a double gene mutation of both *PAX3* and *COL11A2* has not been reported in previous studies. However, we are not sure whether or not these two mutations would have a superimposed effect on hearing damage. We also are not able to compare the severity of hearing loss caused by a single gene mutation with a double gene mutation due to the small sample size.

As a member of the SRY-related HMG-box family, *SOX10* plays an important role in embryonic development, especially in the nervous system, determining the cell fate (Røge et al., 2017). Mutations of *SOX10* may result in either the WS2 or the WS4 (Ahmed jan et al., 2022). Patient 02 was detected to have a nonsense variant of c.426G>A (p.W142X) in *SOX10* gene. Because congenital megacolon and other gastrointestinal disorders were excluded, he was regarded as WS2. However, with clinical findings alone, he would be diagnosed as WS1 due to his dystopia canthorum, which is traditionally noted to be absent from WS2. This conflict illustrates the clinical and genetic heterogeneities that exist in Waardenburg syndrome. Only Patient 03 was diagnosed as WS1 based on his typical characteristics of dystopia canthorum, broad nasal root, synophrys, depigmented iris and fundus. His molecular diagnosis further supported he is WS1 because he carries with a pathogenic variation of c.668G>T (p.R223L) in *PAX3*.

In summary, we present three separate cases of WS with congenital exotropia and find four novel variants in *PAX3*, *COL11A2* and *SOX10* genes. Our finding would expand the mutation spectrum for these three genes. In addition, we find that there is not a widespread association between WS and congenital exotropia, based on our review of the literature. Through genetic experiments, we find that a rare double gene mutation of *PAX3* and *COL11A2* could cause hearing loss in our patient with WS. All mutations detected in *PAX3*, *COL11A2* and *SOX10* are heterozygous variations. As an autosomal dominant disorder, haplotype insufficiency could be the underlying molecular pathogenesis caused by these mutations because these variants are predicted to produce abnormal mRNA and to be degraded by NMD mechanism.

## Data Availability

The datasets for this article are not publicly available due to concerns regarding participant/patient anonymity. Requests to access the datasets should be directed to the corresponding author.

## References

[B1] Ahmed jan N. Mui R. K. Masood S. (2022). Waardenburg syndrome. Tampa, Florida, United States: Treasure Island: StatPearls. Internet. Google Scholar

[B2] Boudewyns A. van den Ende J. Declau F. Wuyts W. Peeters N. Brandt A. H. D. (2020). Etiological work-up in referrals from neonatal hearing screening: 20 Years of experience. Otol. Neurotol. 41 (9), 1240–1248. 10.1097/MAO.0000000000002758 PubMed Abstract | 10.1097/MAO.0000000000002758 | Google Scholar 32925850

[B3] Boudjadi S. Chatterjee B. Sun W. Vemu P. Barr F. G. (2018). The expression and function of PAX3 in development and disease. Gene 666, 145–157. 10.1016/j.gene.2018.04.087 PubMed Abstract | 10.1016/j.gene.2018.04.087 | Google Scholar 29730428 PMC6624083

[B4] Choi Y. M. Kim S. H. (2013). Comparison of clinical features between two different types of exotropia before 12 Months of age based on stereopsis outcome. Ophthalmology 120 (1), 3–7. 10.1016/j.ophtha.2012.07.062 PubMed Abstract | 10.1016/j.ophtha.2012.07.062 | Google Scholar 23031669

[B5] Dastur Y. K. Chitale A. Dasgupta S. DudhAni A. (1995). Waardenburg syndrome with anisocoria and exotropia. J. Postgrad. Med. 41 (4), 111–112. PubMed Abstract | Google Scholar 10707735

[B6] Delleman J. W. Hageman M. J. (1978). Ophthalmological findings in 34 patients with Waardenburg syndrome. J. Pediatr. Ophthalmol. Strabismus 15 (6), 341–345. 10.3928/0191-3913-19781101-03 PubMed Abstract | 10.3928/0191-3913-19781101-03 | Google Scholar 105123

[B7] Farrer L. A. Grudfast K. M. Amos J. Arnos K. S. Asher J. H. Jr Beighton P. (1992). Waardenburg syndrome (WS) type I is caused by defects at multiple loci, one of which is near ALPP on chromosome 2: First report of the WS consortium. Am. J. Hum. Genet. 50 (5), 902–913. PubMed Abstract | Google Scholar 1349198 PMC1682585

[B8] Federico J. R. Krishnamurthy K. (2022). Albinism. Tampa, Florida, United States: Treasure Island (FL): StatPearls. Google Scholar

[B9] Govindan M. Mohney B. G. Diehl N. Burke J. (2005). Incidence and types of childhood exotropia: A population-based study. Ophthalmology 112, 104–108. 10.1016/j.ophtha.2004.07.033 PubMed Abstract | 10.1016/j.ophtha.2004.07.033 | Google Scholar 15629828

[B10] Gowda V. K. Srinivasan V. M. (2020). Waardenburg syndrome type I. Indian J. Pediatr. 87 (3), 244. 10.1007/s12098-019-03170-5 PubMed Abstract | 10.1007/s12098-019-03170-5 | Google Scholar 31989460

[B11] Huang S. Song J. He C. Cai X. Yuan K. Mei L. (2021). Genetic insights, disease mechanisms, and biological therapeutics for Waardenburg syndrome. Gene Ther. (25), 1–19. 10.1038/s41434-021-00240-2 10.1038/s41434-021-00240-2 | Google Scholar 33633356

[B12] Kim H. K. Ankamreddy H. Lee D. J. Kong K. A. Ko H. W. Kim M. H. (2014). Pax3 function is required specifically for inner ear structures with melanogenic fates. Biochem. Biophys. Res. Commun. 445 (3), 608–614. 10.1016/j.bbrc.2014.02.047 PubMed Abstract | 10.1016/j.bbrc.2014.02.047 | Google Scholar 24565836

[B13] Kunst H. Huybrechts C. Marres H. Huygen P. Cremers C. Van Camp G. (2000). The phenotype of DFNA13/col11a2: Nonsyndromic autosomal dominant mid-frequency and high-frequency sensorineural hearing impairment. Am. J. Otol. 21 (2), 181–187. 10.1016/s0196-0709(00)80006-x PubMed Abstract | 10.1016/s0196-0709(00)80006-x | Google Scholar 10733181

[B14] Lueder G. T. Galli M. (2018). Infantile exotropia and developmental delay. J. Pediatr. Ophthalmol. Strabismus 55 (4), 225–228. 10.3928/01913913-20180213-05 PubMed Abstract | 10.3928/01913913-20180213-05 | Google Scholar 29709041

[B15] Read A. P. Newton V. E. (1997). Waardenburg syndrome. J. Med. Genet. 34 (8), 656–665. 10.1136/jmg.34.8.656 PubMed Abstract | 10.1136/jmg.34.8.656 | Google Scholar 9279758 PMC1051028

[B16] Røge R. Nielsen S. Bzorek M. Vyberg M. (2017). NordiQC assessments of SOX10 immunoassays. Appl. Immunohistochem. Mol. Morphol. 25 (6), 377–380. 10.1097/PAI.0000000000000536 PubMed Abstract | 10.1097/PAI.0000000000000536 | Google Scholar 28549040

[B17] Somashekar P. H. Girisha K. M. Nampoothiri S. Gowrishankar K. Devi R. R. Gupta N. (2019). Locus and allelic heterogeneity and phenotypic variability in Waardenburg syndrome. Clin. Genet. 95 (3), 398–402. 10.1111/cge.13468 PubMed Abstract | 10.1111/cge.13468 | Google Scholar 30394532

[B18] Tang X. J. Ping X. Y. Luo C. Q. Yu X. N. Tang Y. L. Shentu X. C. (2020). Dystrophia canthorum in Waardenburg syndrome with a novel MITF mutation. Int. J. Ophthalmol. 13 (7), 1054–1059. 10.18240/ijo.2020.07.06 PubMed Abstract | 10.18240/ijo.2020.07.06 | Google Scholar 32685391 PMC7321951

[B19] Touraine R. L. Attie-Bitach A. Manceau E. Korsch E. Sarda P. Pingault V. (2000). Neurological phenotype in Waardenburg syndrome type 4 correlates with novel SOX10 truncating mutations and expression in developing brain. Am. J. Hum. Genet. 66 (6), 1496–1503. 10.1086/302895 PubMed Abstract | 10.1086/302895 | Google Scholar 10762540 PMC1378013

[B20] Vázquez-Villa F. Garcia-Ocana M. Galván J. A. García-Martínez J. García-Pravia C. Menéndez-Rodríguez P. (2015). COL11A1/(pro)collagen 11A1 expression is a remarkable biomarker of human invasive carcinoma-associated stromal cells and carcinoma progression. Tumour Biol. 36 (4), 2213–2222. 10.1007/s13277-015-3295-4 PubMed Abstract | 10.1007/s13277-015-3295-4 | Google Scholar 25761876

[B21] Verheij J. B. G. M. Hofstra R. M. W. (2016). “EDNRB, EDN3, SOX10, and the shah-waardenburg syndrome (WS4): The molecular basis of clinical disorders of morphogenesis: Epstein's inborn errors of development,” in Epstein's inborn errors of development, 531–535. 10.1093/med/9780199934522.003.0071 | Google Scholar

[B22] Von Noorden G. K. (2002), Binocular vision and ocular motility. Editor Louis S.. 6e ed (Maryland Heights, Missouri, United States: Mosby). Google Scholar

